# The utility of fitness landscapes and big data for predicting evolution

**DOI:** 10.1038/s41437-018-0128-4

**Published:** 2018-08-20

**Authors:** J. Arjan G. M. de Visser, Santiago F. Elena, Inês Fragata, Sebastian Matuszewski

**Affiliations:** 1 Laboratory of Genetics, Wageningen University, Wageningen, The Netherlands; 2 Instituto de Biología Molecular y Celular de Plantas (IBMCP), Consejo Superior de Investigaciones Científicas-Universitat Politècnica de València, València, Spain; 3 Instituto de Biología Integrativa de Sistemas (I2SysBio), Consejo Superior de Investigaciones Científicas-Universitat de València, València, Spain; 4 The Santa Fe Institute, Santa Fe, NM 87501 USA; 5 Instituto Gulbenkian de Ciência, Oeiras, Portugal; 6 Ecole Polytechnique Fédéral de Lausanne, Lausanne, Switzerland

**Keywords:** Evolutionary theory, Evolutionary biology

The prospect that we may be able to predict the outcome of future evolutionary processes has motivated recent investigations of the factors that determine this predictability (Lässig et al. 2017). At first sight, predicting evolution may seem an unsurmountable goal, and perhaps even naïve, given the many stochastic factors involved, prominently among them environmental change and the origin of new heritable variants. Yet, the situation is not entirely hopeless, as we can predict general features of evolution, such as the dynamics of adaptation resulting from available genetic variation (Fisher 1930), and the rate of genome evolution in the absence of selection (Kimura 1968). A particularly complicating factor, realized long ago by Sewall Wright (Wright 1932), is that the fitness consequences of mutations may vary in an unpredictable manner across genetic backgrounds due to pervasive epistasis. To understand evolutionary processes in the face of epistasis, Wright introduced the concept of the fitness landscape. In the visually appealing 3-dimensional version of the fitness landscape, epistasis introduces mountain ranges with multiple peaks, each representing an alternative adaptive solution for a genotype in a particular condition, separated by lower-fitness regions. In one way, such rugged landscapes prevent precise evolutionary predictions, since it is impossible to know towards which of the many peaks evolution will head off or which mutational pathways may be more likely than others. However, Weinreich et al. (2006) emphasized that epistasis also reduces the number of mutational pathways natural selection will promote, thus enhancing predictability once evolution has committed to a particular peak (Palmer et al. 2015). In other words, one may predict the outcome, but not the specific pathway, when epistasis is weak. When epistasis is strong (i.e., the landscape highly rugged) it may be more difficult to predict the outcome, but perhaps the evolutionary pathway can be predicted once the approximate direction of evolution becomes clearer (Szendro et al. 2013; Bank et al. 2016). This realization motivated many recent efforts to analyze fitness landscapes empirically, and to study how their topography directs evolution (de Visser and Krug 2014).

Characterizing the topography of real fitness landscapes by analyzing the interactions among a small subset of mutations is one obvious approach to fill in missing information (de Visser and Krug 2014). Complementary progress comes from theoretical analyses that have provided new tools to characterize these landscapes and explore their evolutionary consequences for varying population-dynamic conditions (Lobkovsky and Koonin 2012; Szendro et al. 2013; Kondrashov and Kondrashov 2015; Ferretti et al. 2016; Zagorski et al. 2016). Nevertheless, the utility of information on fitness landscapes for evolutionary predictions has so far been limited for several reasons. One reason is that empirical studies can only analyze tiny parts of the fitness landscape, leading to potential biases in the inferred topography. For instance, if non-epistatic subsets of mutations are rare, they will be often missed in small-scale empirical analyses, while in reality selection may “find” and “use” them (de Visser and Krug 2014). Another reason is that the environment may change during evolution, including due to changes in the evolving organisms themselves. This may impact the topography of the fitness landscape, and hence limit forward-looking predictions based on the original set of conditions (Mustonen and Lässig 2009). Given these concerns, more promising than predicting specific mutational pathways may be to predict the dynamics of adaptation based on global patterns of epistasis, such as the diminishing returns pattern observed in several organisms (Kryazhimskiy et al. 2014; Martin 2014; Schoustra et al. 2016). Models that can capture these global patterns and predict fitness from a few underlying phenotypes, as for example Fisher’s geometric model (Fisher 1930), may be useful for predicting phenotypic change (Martin et al. 2007; Blanquart et al. 2014; Tenaillon 2014; Blanquart and Bataillon 2016; Hwang et al. 2017). However, despite its utility to predict general patterns of evolution, Fisher’s Geometric model is often unable to explain the real underlying structure of experimental fitness landscapes (Blanquart and Bataillon 2016), making it difficult to obtain predictions that can be applied to current societal problems. In fact, the only studies that have produced predictions with practical utility so far, such as for influenza vaccine selection, have used relatively simple fitness models that did not involve epistasis (Luksza and Lässig 2014; Neher et al. 2016).

This special issue surveys different experimental and theoretical approaches to study the factors that prevent or allow us to predict evolution, motivated by a number of open questions. It originates from the symposium “Fitness landscapes, big data, and the predictability of evolution”, at the European Society for Evolutionary Biology meeting (ESEB XVI) held in August 2017 in Groningen, the Netherlands. The seven studies include a mixture of empirical and theoretical work, often combined in the same study, and together address three global questions: (i) What do real fitness landscapes look like? (ii) How does landscape topography affect evolution? (iii) How do different mutation classes contribute to evolution? Here we highlight their contributions to these questions.

## What do real fitness landscapes look like?

Of the empirically characterized fitness landscapes that are presently available, most concern mutations in a single gene, such as TEM-1 β-lactamase (Weinreich et al. 2006; Schenk et al. 2013) or heat shock proteins (e.g. Hsp90; Hietpas et al. 2011), while a minority involves mutations in different genes for a variety of micro-organisms, which often have been co-selected in the same genetic background (Khan et al. 2011; Lalić and Elena 2015). However, recent methods to systematically generate mutants and measure their fitness effects in bulk competitions using deep sequencing (Hietpas et al. 2011) now allow analyses of landscapes involving many thousands of genotypes (e.g., Acevedo et al. 2014). Analyses of these empirical fitness landscapes have already yielded a few general, but preliminary insights (de Visser and Krug 2014). One general finding is that real fitness landscapes are rugged, but that the level of ruggedness varies substantially across studies. A few factors have been identified that seem to underlie this variation by increasing the strength of epistasis. These include the fitness effect of mutations, their occurrence in the same rather than different genes, and the fact that the collective effects of the mutations involved were unknown a priori. These insights will help understanding of the variation in topography of the growing collection of empirical landscapes. Other studies have explicitly measured landscape topographies under varying conditions, including for five non-synonymous mutations in a long-term experiment with *Escherichia coli* (Flynn et al. 2013), combinations of a transcription factor and operator of the *lac* operon (de Vos et al. 2015), and for random synonymous and non-synonymous mutations in an RNA plant virus tested on different host species (Lalić and Elena 2012; Cervera et al. 2016a). All these studies show that a plethora of factors can influence the topography of fitness landscapes.

Interestingly, two studies in this special issue now provide the first insights that synonymous mutations can also influence fitness landscape topography. Zwart et al. present an empirical fitness landscape involving four synonymous mutations that individually increase the activity of TEM-1 β-lactamase on a novel substrate, the antibiotic cefotaxime. They show surprisingly strong epistatic interactions among these mutations, particularly given the relative small effect of the mutations (refuting the idea that the strength of epistasis scales with the fitness effect-size of the individual mutations). Zwart et al. then use their empirical fitness landscape to show that these synonymous mutations render their benefit via affecting more than one phenotype. Similarly, for the yeast chaperone Hsp90, Fragata & Matuszewski et al. show that the impact of synonymous mutations on the topography of the fitness landscape is environment dependent. In addition to finding clear fitness effects of synonymous mutations, these studies show how analyses of their interactions may help to identify the mechanisms responsible.

## How does landscape topography affect evolution?

Empirical tests of predictions about evolutionary trajectories or outcomes based on fitness landscape analyses are scarce. For example, Salverda et al. (2011) tested, and partly confirmed, Weinreich et al. (2006) prediction that TEM-1 β-lactamase adapts to the antibiotic cefotaxime by using a specific set of four amino-acid substitutions in strict order. In sharp contrast, Cervera et al. (2016b) observed that evolving genotypes of tobacco etch potyvirus (TEV) located at increasing distances from a local fitness peak did not recapitulate the expected evolutionary path but explored other regions of the genotypic landscape. Generally, deviations from such predictions may have multiple causes, including contributions of other mutations outside of the small subset considered for the empirical landscape, and evolutionary changes in the selective conditions that result in changes in the topography. These fundamental problems have been addressed in at least two distinct ways. First, instead of testing a priori predictions from empirical fitness landscapes, evolution experiments have been combined with *a* posteriori analyses of interactions among common mutations to test the role of epistatic constraints in directing the pathways observed. This approach was used to understand the higher evolvability of a low-fitness genotype in a long-term experiment with *E. coli* (Woods et al. 2011), and in another study to understand distinct mutational paths during yeast evolution to slow and rapid increases of nickel (Gorter et al. 2018). Gifford et al. now add a study in which results from evolution and mutation accumulation experiments with bacteria are combined with simulations to address the relative contribution of standing genetic variation and de novo mutations to the evolution of antibiotic resistance at different drug concentrations. Most interestingly, they found that altering the nature of environmental pleiotropy also alters the relationship between a mutant’s frequency and its fitness. When fitness values correlate positively across antibiotic concentrations, selection for the strongest resistance mutations is reinforced; in contrast, when uncorrelated effects reduced the strength of selection.

A different approach is to explore, and try to predict, evolution on (empirical or model) fitness landscapes using theoretical models. This special issue contains three contributions of this kind. McCandlish develops a mathematical framework for understanding evolution on fitness landscapes when mutations are rare. In contrast to standard assumptions, McCandlish’s approach does not require selection to be strong and allows deleterious mutations to become fixed – adding biological realism to these types of models. The resulting reversible Markov-chain model is then used to classify parts of the fitness landscape (and the mutations defining it) by their “mutational” and “dynamic neighborhoods” – characterizing regions of the fitness landscape that are easy or rather difficult to reach due to mutational distance and the presence of a fitness valley. These two quantities shed surprising light on the structure of the fitness landscape – particularly where the mutational and dynamical neighborhood of a genotype are misaligned.

Similarly, Ferretti et al. propose new statistical measures for quantifying the evolutionary constraints on both theoretical and empirical fitness landscapes: the similarity between accessible paths and the abundance and characteristics of “chains of obligatory mutations”; i.e., paths of genotypes with only a single beneficial mutation available. Unlike most conventional measures of epistasis, or landscape ruggedness, they tend to be only weakly correlated with one another, but also with the strength of epistasis. Interestingly, Ferretti et al. show that the number of “chains of obligatory mutations” is maximal for intermediately rugged fitness landscapes, emphasizing that it captures information about the structure of epistasis, and thus could represent an evolutionarily meaningful tool to classify and discriminate between different types of fitness landscapes.

Finally, Passagem-Santos et al. compared the ability of several phenotype-fitness models to predict adaptive dynamics, using both simulations and empirical data from *E. coli* and *Schizosaccharomyces pombe*. This study adds to the growing literature of empirical studies that focus on how general and simple phenotype-fitness models, such as the power-law, Fisher’s Geometric Model or the stickbreaking model (Wiser et al. 2013), can predict long-term adaptation to a single environment. Interestingly, Passagem-Santos et al. argue that despite their simplicity, these models can already capture the patterns of diminishing-returns epistasis. They allow mimicking of the pattern of decelerating adaption (i.e., as populations approach a fitness optimum, adaptation proceeds through smaller mutational steps, Martin et al. 2007, Khan et al. 2011, Draghi and Plotkin 2013), particularly in a constant environment, and suggests that it is possible to predict general features of adaptation in laboratory evolution experiments.

## How do different mutation classes contribute to evolution?

An important and elegant prediction from population genetic theory is that the substitution rate is equal to the mutation rate, as long as selection is unimportant and mutations all arise via single mutant individuals (Kimura 1968). The latter implies that mutational bias (i.e., the variation in frequency at which different mutations arise), has a major impact on genome evolution. Under selection, beneficial mutations will substitute disproportionally as a function of their fitness benefit, but mutational bias will still play a role, as small-benefit mutations (that frequently occur) may substitute before less frequent higher-benefit mutations (e.g., Cooper et al. 2001). In this issue, Willemsen et al. want to better understand one particular source of mutational bias, genome deletions, by analyzing the stability of genomic insertions in TEV. They find that the recombination rate is the best predictor of insertion stability, as recombination is a dominant mechanism of insert deletions. As described above, Zwart et al. and Fragata & Matuszewski et al. specifically address the evolutionary impact of another class of mutations, namely synonymous mutations. Both studies find significant positive fitness effects of these mutations, and identify possible underlying mechanisms explaining these effects: post-transcriptional positive effects on enzyme levels for the β-lactamase mutations in *E. coli* reported by Zwart et al., and residue position, mRNA stability and codon frequency for the mutations in Hsp90 of yeast by Fragata & Matuszewski et al. Together, these studies make a strong case for why the evolutionary contribution of synonymous mutations and genome deletions should not be neglected.

## Future perspectives

This special issue highlights several remaining open questions, whose answers may guide future work on evolutionary predictions:

1. To what extent is the fitness landscape concept useful for understanding and predicting evolution? Do we need to move away from the present static paradigm to dynamic landscapes that incorporate environmental changes (Mustonen and Lässig 2009; Catalán et al. 2017)?
2. Which topological features of fitness landscapes are most informative to allow evolutionary predictions? E.g. general features such as average ruggedness, number of peaks, pervasiveness of higher-order epistasis, or more local features such as “chains of obligatory genotypes” (Ferretti et al.)?
3. What models should we build to capture these topological features? It is clear that full fitness landscapes cannot be empirically characterized, so we need models to extrapolate partial empirical data into full landscapes. Two approaches have been used so far: (i) bottom-up biophysical models, capturing real molecular interactions at play (e.g., DNA-transcription factor binding); and (ii) statistical models (e.g., Rough Mount Fuji or NK) that describe global statistical features, such as ruggedness, or peak number. How informative is each class of models for evolutionary predictions?
